# Editorial: Sphingolipids in infections, diseases, and disorders

**DOI:** 10.3389/fimmu.2024.1392055

**Published:** 2024-03-14

**Authors:** Farha Naz, Mohd Arish, Imtaiyaz Hassan

**Affiliations:** ^1^ Department of Medicine, Division of Infectious Diseases and International Health, University of Virginia School of Medicine, Charlottesville, VA, United States; ^2^ Carter Immunology Center, University of Virginia School of Medicine, Charlottesville, VA, United States; ^3^ Centre for Interdisciplinary Research in Basic Sciences, Jamia Millia Islamia, New Delhi, India

**Keywords:** sphingosine 1-phosphate (S1P) receptors, sphingosine 1-phosphate (PubChem CID: 5283560), sphingolipid-based approaches, S1PR modulators, infectious diseases

Sphingosine 1-phosphate (S1P) signaling is a critical regulator of myriads of cellular functions and operates through a family of G protein-coupled receptors known as S1P receptors (S1PR1-5) (1). This signaling pathway influences fundamental processes such as cell proliferation, migration, angiogenesis, and immune responses. Dysregulation of S1P signaling has been implicated in various diseases, including cancer, autoimmune disorders, cardiovascular conditions, and infectious diseases (2, 3). Targeting S1P signaling has emerged as a promising therapeutic approach for these pathologies (3, 4).

S1P is one of the products of sphingolipid metabolic pathways. This *de novo* synthesis pathway has been extensively reviewed by Jamjoum et al. The authors summarized that this pathway occurs on the cytosolic surface of the endoplasmic reticulum and initiates with the condensation of L-serine and palmitoyl-CoA to produce a series of sphingolipids metabolites such as sphinganine, ceramide, and sphingosine (5). S1P biogenesis requires a sphingosine kinase that phosphorylates sphingosine into S1P. The expression of SK1 also determines the dysregulation of S1P signaling. For instance, this kinase has been reported to be downregulated in macrophages upon various bacterial and parasitic infections (6, 7). It was also reported that S1PR modulators such as S1PR2-3 can limit intracellular mycobacterial load by polarizing human macrophages into M1 phenotype (8). To this end, Mohammed et al in this Research Topic has further advocated sphingolipid-based approaches to mitigate Tuberculosis. They have further suggested overexpression of host sphingomyelinase activity, which helps in the elevation of ceramide levels that could reprogram macrophages to promote acidification of phagolysome for eliminating mycobacterial burden (9). Also, the authors concluded that elevated ceramide may be converted to S1P and help to further differentiation of infected macrophages into mycobactericidal M1 macrophages.

Some studies suggest that SK1 may facilitate viral entry and replication. For example, SK1 activity has been shown to promote the entry of certain viruses into host cells, including human immunodeficiency virus (HIV) and hepatitis C virus (HCV) (10). Increased SphK1 activity has been associated with liver injury and fibrosis in HCV infection (11). In the context of COVID-19, it was assumed that S1P signaling may provide adjunctive therapy to battle SARS-Cov-2-induced hyper-inflammatory response in the lungs of patients (12, 13). To further support this, a study by Marfia et al. showed decreased S1P serum levels in infected patients, which also serve are a biomarker of disease severity (14). In contrast, Khan et al found that SK1 expression was increased in COVID patients’ lungs, which was further enriched in Type II pneumocytes and alveolar macrophages (15). However, the study conducted by Khan et al was focused on the lung, the site of infection, and also the expression of SK1 was limited to alveolar macrophages and type II cells.

S1P receptors (S1PRs) are modulated by various agents, including agonists and antagonists, which can either activate or inhibit their signaling pathways. S1PR modulation is one of the key strategies for harnessing S1P signaling (8). Some of the modulators are in preclinical and clinical trials for various diseases. However, modulators such as Fingolimod (Gilenya) and Siponimod (Mayzent) have already been approved by the FDA for the treatment of Multiple Sclerosis, a chronic disease of the central nervous system. Nevertheless, it is highly anticipated that more S1P modulators can be repurposed for the treatment of other diseases in the coming years. One such contribution was from Li et al study where the group demonstrated that the sphingosine-1-phosphate receptor 1 (S1PR1) modulator, ozanimod, improves microvascular hemodynamics after cerebrovascular thrombosis in the mouse. Furthermore, ozanimod can also improve the thrombolytic effect of a sub-thrombolytic dose of issue-type plasminogen activator (tPA) only FDA-approved treatment of acute ischemic stroke (AIS) (16).

The recent articles compiled under this Research Topic have brought to light the crucial role of S1P signaling in the development and progression of various diseases and disorders. Moreover, these articles have emphasized the immense potential of S1PR modulators in designing innovative and highly effective treatment regimes against such ailments. The findings presented in these articles are a testament to the significant impact that S1P signaling and S1PR modulators can have in the field of medical research and healthcare.

## Author contributions

FN: Writing – original draft, Writing – review & editing. MA: Writing – original draft, Writing – review & editing. IH: Writing – review & editing.
